# Association of Nicotine with Osteochondrogenesis and Osteoarthritis Development: The State of the Art of Preclinical Research

**DOI:** 10.3390/jcm8101699

**Published:** 2019-10-16

**Authors:** Xiaoyu Cai, Liang Gao, Magali Cucchiarini, Henning Madry

**Affiliations:** 1 Center of Experimental Orthopaedics, Saarland University Medical Center and Saarland University, 66421 Homburg/Saar, Germany; xiaoyucai0418@gmail.com (X.C.); liang.gao@uni-saarland.de (L.G.); mmcucchiarini@hotmail.com (M.C.); 2 Department of Orthopaedic Surgery, Saarland University Medical Center and Saarland University, 66421 Homburg/Saar, Germany

**Keywords:** systematic review, smoking, nicotine, cigarette, cartilage, articular cartilage, chondrocyte, osteoarthritis

## Abstract

The deleterious effects of nicotine on various health conditions have been well documented. Although many orthopedic diseases are adversely affected by nicotine, little is known about its preclinical effects on chondrogenesis or osteogenesis, cartilage formation, osteoarthritis (OA), and osteochondral repair. A systematic review was conducted examining the current scientific evidence on the effects of nicotine on chondrogenesis or osteogenesis in vitro, possible consequences of prenatal nicotine exposure (PNE) on cartilage and OA susceptibility in the offspring, and whether nicotine affects OA development and osteochondral repair in vivo, always focusing on their underlying mechanisms. The data reveal dose-dependent effects on articular chondrocytes and on the chondrogenesis and osteogenesis of medicinal signaling cells in vitro, with lower doses often resulting in positive effects and higher doses causing negative effects. PNE negatively affects articular cartilage development and induces OA in the offspring without or with nicotine exposure. In contrast, protective effects on OA development were only reported in monosodium iodoacetate-induced small animal models. Finally, nicotine repressed MSC-based osteochondral repair in vivo. Future studies need to investigate dose-dependent clinical effects of smoking on cartilage quality in offspring, OA susceptibility and progression, and osteochondral repair more in detail, thus identifying possible thresholds for its pathological effects.

## 1. Introduction

Articular cartilage, the gliding tissue that covers the ends of bones, has a limited capacity to repair [1]. Osteoarthritis (OA) and focal (osteo-)chondral defects caused by trauma or osteochondral diseases, such as osteochondritis dissecans are often debilitating conditions chiefly affecting the articular cartilage, besides the subchondral bone and all other tissues forming diarthrodial joints [2,3,4]. Nicotine, the principal pharmacologically active component of smoking tobacco, smokeless tobacco, and e-cigarettes, has been long recognized as an important influencer of cartilage formation and OA [5,6,7,8,9].

Although many musculoskeletal conditions are complicated and adversely affected by cigarette smoking, including fracture repair and osteoporosis, spinal fusion, and degenerative disc disease, divergent data exist on the precise effects of nicotine on osteochondrogenesis and OA development due to the diversity of previous evidence from both preclinical and clinical trials [10,11,12,13,14]. For example, several epidemiological studies support the hypothesis that smoking may prevent OA [5,6,12,15,16]; however, preclinical studies indicate that prenatal nicotine exposure (PNE) suppressed insulin-like growth factor I (IGF-I) expression in fetal articular chondrocytes and down-regulated the chondrogenic differentiation of bone marrow-derived mesenchymal stem cells [9,17]. In the following, the term “medicinal signaling cells” (MSCs) will be used when referring to either mesenchymal stem or stromal cells [18]. Although original reports on potential complications of nicotine or smoking on articular cartilage and knee OA have increased in recent years, a systematic analysis of the currently available basic research has, to the best of our knowledge, not yet been performed. As the nicotine and smoking dosage could be converted interspecies according to the average body surface area and species-specific correction factors, a systematic analysis may be important for translating preclinical evidence to the clinical situation.

Here, we aim to provide an analytical summary of the current preclinical evidence regarding the association of nicotine/smoking with osteochondrogenesis, osteochondral repair, and OA development. The following four questions were specifically addressed: (1) Which effects does nicotine have on the chondrogenesis or osteogenesis of MSCs and articular chondrocytes in vitro? (2) Does PNE have an effect on cartilage and OA susceptibility in the offspring? (3) Does nicotine/smoking affect OA development and progression and osteochondral repair in animal models in vivo? and (4) What are the mechanisms of those effects of nicotine/smoking on the osteochondrogenesis and OA development?

## 2. Materials and Methods

### 2.1. Search Strategy

A systematic literature search was performed within the PubMed (1966–2019.09) and ScienceDirect (1985–2019.09) databases, using the search terms “smoking”, “smokeless tobacco”, “vaping”, “e-cigarette”, “nicotine”, “cartilage”, and “osteoarthritis”. Studies were enrolled investigating associations between the nicotine/tobacco/smoking/vaping and osteochondrogenesis, osteochondral repair, or OA development. Clinical trials, reviews, and non-English articles were excluded. Two reviewers (L.G. and X.Y.C.) independently screened all articles, and disagreement was resolved by consultation with a third reviewer (H.M.). All abstracts and titles of the studies were initially screened. Full texts were then obtained for all studies meeting the inclusion criteria and were reviewed to reconfirm their eligibility.

### 2.2. Data Extraction and Critical Appraisal

The data extracted included nicotine dose and cell/animal type, treatment groups, follow-ups, measures, and main outcomes. Data were collected by one reviewer in a standardized extraction form and verified by the other two reviewers to reach a consensus. Due to the methodological heterogeneity of the outcome assessment in the included studies, only data from outcome measures with proven validity and reliability were further selected and aggregated.

## 3. Results

### 3.1. Search Results and the Characteristics of the Included Studies

**Figure 1:**
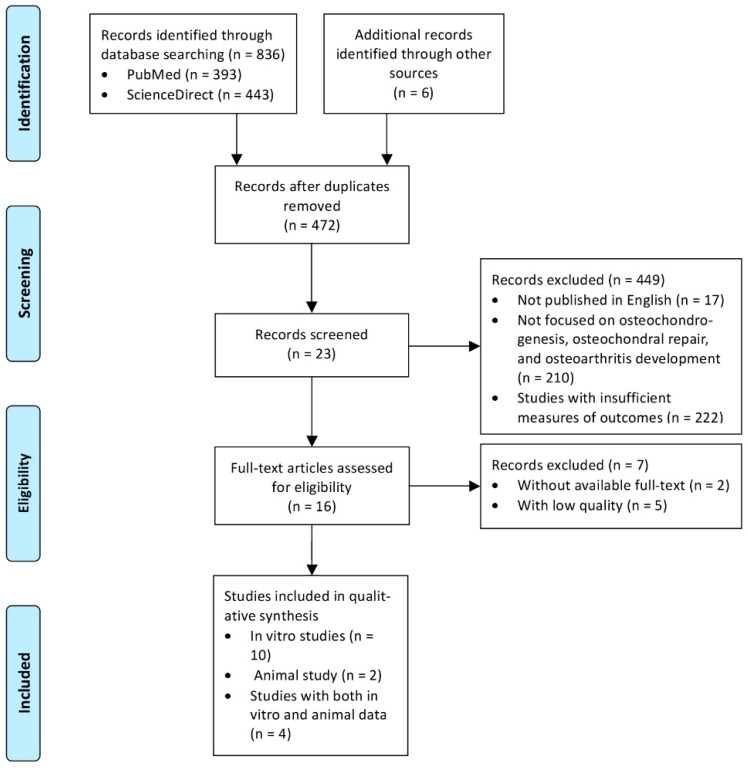
Study selection progress according to the Preferred Reporting Items for Systematic Reviews and Meta-Analyses guidelines [30].

A total of 842 papers were initially identified. After the removal of duplicates, 472 articles fulfilled the inclusion criteria (Figure 1). After screening of their titles, abstracts, and full texts with the aforementioned criteria, 16 articles were finally included: 14 in vitro studies regarding effects of nicotine or smoke extract on MSCs osteochondrogenesis (Table S1) [9,19,20,21,22,23,24,25], osteoblast differentiation (Table S2) [26,27], and chondrocyte proliferation (Table S3) [7,12,17,28] and six animal studies regarding the effects of nicotine on osteochondral repair [9] and OA progression (Table S4) [12,15] and the outcomes of PNE on cartilage formation [28] and OA susceptibility in the offspring (Table S5) [17,29].

### 3.2. In Vitro Studies

#### 3.2.1. In Vitro Studies on the Effect of Nicotine on Chondrogenic Differentiation of Medicinal Signaling Cells

##### Bone Marrow-derived MSCs

Ying et al. reported the effects of nicotine (0, 0.1, 1, and 10 μM) on the proliferation and chondrogenesis of human bone marrow-derived (BM) MSCs (BM-MSCs) [22]. Cell viability was not significantly impaired in all nicotine groups except for 10 μM nicotine. Cell proliferation was increased significantly with both 0.1 and 1 μM nicotine; however, it was inhibited significantly with 10 μM nicotine. Expression of type-II collagen mRNA and protein was significantly up-regulated with 0.1 and 1 μM nicotine, while the expression of aggrecan mRNA was significantly reduced with 10 μM nicotine. Altogether, the data show a dose-dependent effect of nicotine on the expression of chondrogenesis-related genes.

Deng et al. explored the possible adverse effects of high-dose nicotine (25, 50, and 100 μM) on chondrogenesis of rat BM-MSCs [23]. Glycosaminoglycan content was significantly reduced in an inverse dose-dependent manner following nicotine treatment. Expression of aggrecan, type-II collagen, and IGF-I mRNAs was significantly reduced, indicating that high-doses of nicotine suppress the chondrogenic potential of BM-MSCs.

Tie et al. also studied short-term effects of nicotine (0.1, 1, 10, and 100 μM) on chondrogenic differentiation of rat BM-MSCs [9]. Expression of sex determining region Y-type high mobility group box 9 (SOX9) mRNA and protein was significantly decreased following nicotine treatment. Expression of nucleic nuclear factor of activated T cells (NFATc)-2 protein was significantly elevated and the cytoplasm phosphorylated NFATc2 was significantly reduced. Additionally, the results also showed no effect on the expression of SOX9 when 10 μM methyllycaconitine (MLA) and a short interfering RNA of NFATc 2 were applied before the nicotine exposure. The data suggest that a nicotine-induced suppression of SOX9 was initiated via activating the Ca^2+^/calcineurin/NFATc2 signaling pathway through α7-nicotine acetylcholine receptor (nAChR).

##### Adipose-Derived MSCs

Wahl et al. investigated the effect of cigarette smoke extract (CSE) (V/V%; 0, 0.5, 1, 5, and 10%) on migration and chondrogenesis of human adipose-derived MSCs (AD-MSCs) [19]. 0.5% and 1% CSE-treated cells exhibited continual migration without significantly altered cell viability, 5% CSE-treated cells showed reduced viability, while 10% CSE-treated cells scarcely survived. 0.5% and 1% CSE did not significantly alter the metabolic activity and survival of AD-MSCs except at 5% and 10% CSE. Angiogenic cytokines were significantly inhibited with 0.5% CSE. AD-MSCs treated with 0.5% CSE had a significantly higher expression of aggrecan and SOX9 mRNAs with reduced mechanical function. In summary, high-dose CSE (> 5%) exposure drastically impaired the cell migration and viability, while low-dose CSE (0.5%) inhibited chondrogenesis of AD-MSCs.

##### Wharton’s Jelly-Derived MSCs

Yang et al. investigated the chondrogenesis of human Wharton’s jelly-derived MSCs (WJ-MSCs) treated with 5 μM nicotine [24]. Nicotine treatment significantly impaired the proliferation of WJ-MSCs without alteration of cellular viability. Significantly down-regulated expression of mRNAs (SOX9, type-II collagen, and aggrecan) and stimulated expression and functional activity of α7-nAChR was observed at day 28. These data indicate that impaired cellular proliferation and chondrogenesis might attribute to the functional activity of α7-nAChR with subsequent calcium influx stimulated by the high-dose nicotine in WJ-MSCs.

#### 3.2.2. In Vitro Studies on the Effects of Nicotine on Articular Chondrocytes

Ying et al. reported the effects of nicotine (0, 0.15, 0.3, and 0.6 μM) on either normal human or OA knee articular chondrocytes [7]. Nicotine significantly increased cell proliferation of both groups in a concentration- and time-dependent manner. The level of type-II collagen mRNA was significantly up-regulated in nicotine-treated chondrocytes compared with the controls. Type-II collagen and aggrecan protein were significantly higher after nicotine treatment compared with controls in both groups; however, significantly less in human OA chondrocytes.

Tie et al. investigated the effects of nicotine (0, 0.4, 2, and 10 μM) on neonate rat articular chondrocytes [17]. Both α4- and β2-nAChR were expressed after 10-day treatment. The Col2a1 mRNA, aggrecan mRNA, and glycosaminoglycan protein increased significantly in a concentration-dependent manner. Th expression of catabolic genes was significantly and concentration-dependently suppressed by nicotine. The expression of mRNAs of the IGF-I signaling pathway was significantly decreased in a concentration-dependent manner by nicotine. Dihydro-β-erythroidin, a selective α4β2-nAChR inhibitor, rescued these deleterious effects induced by 10 μM nicotine, suggesting that α4β2-nAchR mediates the effects of nicotine on articular chondrocytes.

Xie et al. investigated the effect of nicotine (0.1, 1, 10, and 100 μM) on transforming growth factor-beta (TGF-β) signaling and extracellular matrix (ECM) expression in normal neonatal rat articular chondrocytes [28]. Nicotine (1, 10, and 100 μM) significantly inhibited the SOX9, Col2a1, and aggrecan mRNAs expression without affecting cell proliferation and mRNA expression of the TGF-β signaling pathway. Additionally, 0.1 μM nicotine significantly reduced Col2a1 and aggrecan mRNAs. Corticosterone (250, 500, and 1250 nM) induced lysine 9 of histone H3 (H3K9) deacetylation at TGF-βR1 and Col2a1 promoters; however; 100 μM nicotine did not alter the H3K9 acetylation. These data show that nicotine does not inhibit the TGF-β signaling pathway directly and that the PNE-induced suppression of TGF-β axis may be mediated by corticosterone.

Liu et al. investigated the effects of 10 μM nicotine on p38, extracellular signal-regulated kinase (ERK) 1/2, and c-Jun-N-terminal kinase (JNK) mitogen-activated protein kinases (MAPK) phosphorylation induced by monosodium iodoacetate (MIA), and IL-1β in adult normal rat chondrocytes [12]. MIA and IL-1β treatment significantly induced rapid and transient phosphorylation of p38, ERK1/2 and JNK. 10 μM nicotine pretreatment significantly attenuated MIA- and IL-1β-induced phosphorylation’s, which could be prevented by MLA.

#### 3.2.3. In Vitro Studies on the Effect of Nicotine on Osteogenic Differentiation of MSCs

##### Bone Marrow-Derived MSCs

Ng et al. investigated the effects of 1 μM nicotine on the cell proliferation, migration, and osteogenesis of human BM-MSCs [20]. Calcium deposition and alkaline phosphatase (ALP) content of MSCs were reduced significantly under osteogenic differentiation with a 10-day nicotine treatment. Osteogenesis-related and cell migration-related gene expression was significantly down-regulated following the nicotine treatment, suggesting that nicotine inhibits osteoblastic differentiation of BM-MSCs.

Shaito et al. studied the effects of e-cigarette smoke extract on osteogenic differentiation of human BM-MSCs, demonstrating that the smoke extract significantly decreased type-I collagen and Runx2 expression, altered cellular morphology with less mineralization, and generated more reactive oxygen species [25].

##### Periodontal Ligament-Derived Signaling Cells

Ng et al. also investigated similar studies with human periodontal ligament-derived signaling cells (PDLSCs) [20]. Cell proliferation and migration of 1 μM nicotine-treated PDLSCs were significantly reduced. Osteogenesis-related miRNAs were significantly up-regulated following nicotine treatment. Subsequent gene expression analysis identified significantly down-regulated expressions of osteogenesis-related genes, further supporting the inhibitory effect of nicotine on PDLSCs osteogenic differentiation potential.

Ng et al. further studied the osteogenic potential of PDLSCs harvested from cigarette smokers [21]. Cell proliferation and migration were significantly reduced compared with non-smokers. Calcium deposition and ALP activity were significantly reduced in PDLSCs from smokers with induced osteogenesis differentiation. Furthermore, nicotine-related miRNAs were significantly up-regulated in PDLSCs from smokers, suggesting their possible important roles in the deteriorative effects on PDLSCs by cigarette smoke.

##### Adipose-Derived MSCs

Wahl and colleagues investigated the effect of CSE exposure on the osteogenesis of AD-MSCs [19]. The osteogenic differentiation was reduced with the inhibited expression of early osteogenic makers after a three-day 0.5% CSE exposure, but it was gradually recovered after a one-week period with significantly increased expression of the late osteoblastic marker osteocalcin, suggesting that osteogenesis inhibited by low-dose CSE may be recovered over time.

#### 3.2.4. In Vitro Studies of Effects of Smokeless Tobacco Extract (STE) on Osteoblast Differentiation

Lenz et al. studied the effects of smokeless tobacco extract (STE) on osteoblasts obtained from chick embryo clavarias and found that STE irreversibly inhibited osteoblastic differentiation with decreased bone collagen synthesis [27]. Henderson et al. showed that diluted STE influenced osteoblast proliferation, differentiation, and metabolism in a dose-dependent manner [26].

### 3.3. Animal Studies

Six animal studies were identified investigating possible effects of PNE or nicotine on articular cartilage development and OA susceptibility in offspring. Deleterious effects of PNE were reported on cartilage quality and the OA susceptibility in adult offspring. Additionally, the animal studies demonstrated that nicotine negatively influenced osteochondral defect repair and suppressed MIA-induced cartilage degradation in a dose-dependent fashion.

#### 3.3.1. Effect of Nicotine on Cartilage Formation in the Offspring

Xie et al. showed that PNE intergenerationally programs impaired articular cartilage via histone deacetylation through maternal lineage [28]. Significantly impaired formation of cartilage in both PNE-F1 and PNE-F2 offspring, reduced ECM expression, and weakened TGF-β signaling in adults were observed. In both neonatal and adult offspring, significantly inhibited acetylation of H3K9 gene promoters was detected. Fewer cells and significantly reduced extracellular matrix staining was noticed within the articular cartilage of all offspring with reduced Col2a1 expression. In the adult PNE-F2 offspring, the deacetylation at H3K9 of the TGF-βR1 and Col2a1 promoters, directly induced by corticosterone (rather than nicotine), might be involved in the diminished cartilage formation in adult PNE-F2 offspring.

#### 3.3.2. Effect of Nicotine on OA Susceptibility in Offspring

Tie et al. demonstrated the effects of PNE on rat OA susceptibility after strenuous running in adult F1 offspring following subcutaneous injections of nicotine during the gestational days 11–20 [17]. Interestingly, birth weight was significantly decreased, and the intrauterine growth retardation rate was significantly increased in the PNE group. Likewise, significantly elevated serum corticosterone and reduced IGF-I concentrations were observed in the PNE neonates. Expression of IGF-I, serine-threonine protein kinase ½, and SOX9 protein was significantly reduced in PNE neonates and adult offspring. Expression of Col2a1 mRNA and protein was significantly decreased in both PNE and control groups. The data revealed that PNE impeded the expression of the SOX9, Col2a1, and IGF-I signaling pathways in articular cartilage of offspring with increased OA susceptibility. By qualitative macroscopic examination, knee joints from the PNE group with strenuous running showed more cartilage erosions than the control group, while PNE had no effect under nonrunning conditions. Histological scoring was significantly worse in the PNE group with strenuous running compared with the normal control group, while no effects under no running conditions were seen. Moreover, significant less Col2a1 was present in the cartilage of the PNE rats in both the no running and running groups, suggesting that PNE inhibits the synthesis of type-II collagen and increases the susceptibility of adult offspring to OA when challenged.

Tie et al. also evaluated the articular cartilage of adult rat offspring fed with a high-fat diet from rats intragestationally treated with subcutaneous nicotine injection [29]. Significantly increased IGF-I, total cholesterol, and low-density lipoprotein cholesterol, and decreased corticosterone levels were observed in the PNE group. Significantly decreased expression of Col2a1 mRNA in neonates and expression of IGF-I, liver X receptor β, adenosine triphosphate-binding cassette transporter A1 and Col2a1 in both neonates and adult offspring were reduced in the PNE group. Significantly worse histological scores were observed in the PNE group. Thus, PNE induced impaired articular cartilage quality in adult offspring and increased OA susceptibility.

#### 3.3.3. Effect of Nicotine in Animal Models of OA

Liu et al. investigated the effects of nicotine on MIA-induced articular cartilage degradation in adult Sprague-Dawley rats treated with intraperitoneal nicotine injections [12]. Interestingly, nicotine reduced macroscopic and histological indices of joint degradation, although the data also implied that nicotine did not fully prevent MIA-induced cartilage degeneration. Administration of the α7-nAChR-selective antagonist methyllycaconitine (MLA) abolished these protective effects. Teng et al. reported the effects of nicotine on MIA-induced cartilage degradation in adult mice treated with intraperitoneal nicotine injection [15]. Nicotine significantly attenuated OA-induced mechanical allodynia in a dose-dependent manner. However, MLA significantly reversed the analgesic effect of nicotine. Nicotine significantly reduced cartilage degradation also in a dose-dependent manner and the upregulation of matrix metalloproteinase 9 (MMP-9), a hallmark of joint inflammation in OA. The activities of MMP-9 were significantly increased by MIA treatment and reduced by nicotine treatment. Furthermore, MLA pretreatment blocked the suppressive effect of nicotine on the MMP-9 expression. The data of these 2 studied suggest that nicotine suppresses MIA-induced cartilage degradation in mice and rats by activating the α7-nAChR.

#### 3.3.4. Effect of Nicotine on Osteochondral Repair

Tie et al. investigated the effect of nicotine on the repair of focal osteochondral trochlear defects in adult rats treated with BM-MSCs encapsulated in alginate [9]. Postoperative subcutaneous nicotine injections yielded significantly worse macroscopic and histological scores of osteochondral defect repair. Reduced expression of Col2a1, aggrecan, and SOX9 mRNAs was observed, possibly related to the negative influence of nicotine on the osteochondral repair.

## 4. Discussion

This systematic review provides a summary of the current preclinical evidence of the effects of nicotine/smoking on MSCs and chondrocytes in vitro and OA development and osteochondral repair in vivo. Altogether, the data show that nicotine has dose-dependent effects on both articular chondrocytes and on the chondrogenesis and osteogenesis of MSCs in vitro, with lower doses often leading to positive effects while higher doses causing negative effects in different in vitro models. PNE negatively affects articular cartilage development and induces OA in offspring without or with nicotine exposure in small animal models. In contrast, protective effects on OA development were only reported in MIA-induced small animal models. Finally, nicotine repressed MSC-based osteochondral repair in vivo.

Nicotine has a dose-dependent effect on chondrogenic differentiation of MSCs and articular chondrocytes in vitro and on cartilage formation in animal models. Such a dose-dependence describes the magnitude of the response of an organism, as a function of doses to a stimulus or stressor after a certain exposure time [31,32]. In vitro studies demonstrated the dose-dependent effect of nicotine on articular chondrocytes and MSCs [7,17,28]. However, the dose-dependent effects were seen in a time-dependent manner [9,19,22,23,24]. In animal models, Xie et al. reported that nicotine suppressed the formation of cartilage in adults of PNE-F1 and PNE-F2 offspring [28]. As only one concentration was tested, more translational in vivo studies are warranted to further investigate the effects of different dose ranges of nicotine on articular cartilage formation.

**Figure 2:**
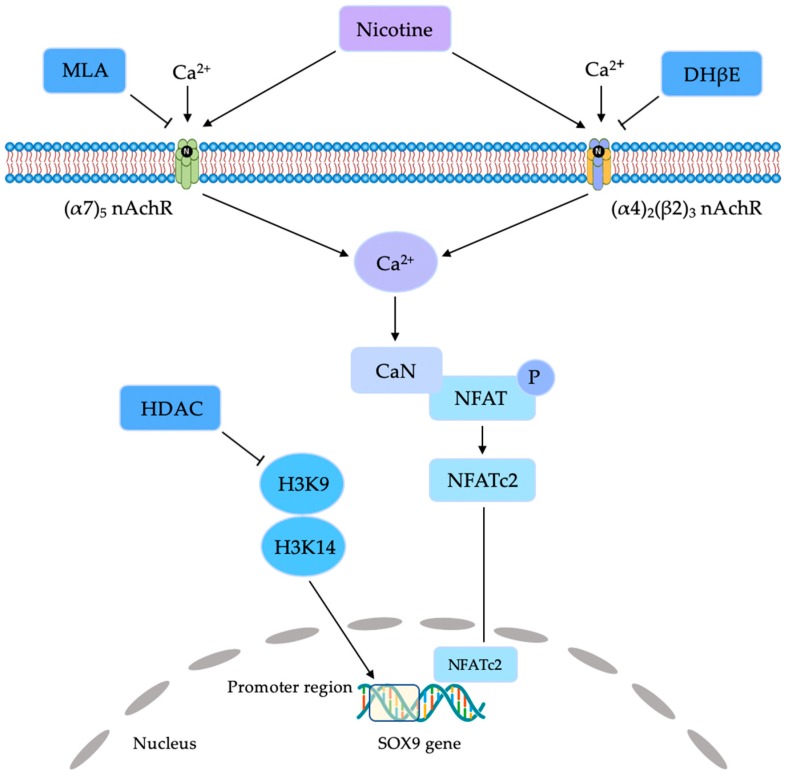
Schematic of nicotine-mediated signaling pathway with the stimulation of (α7)5 nicotine acetylcholine receptor (nAchR) and (α4)2(β2)3 nAchR. CaN, Calcineurin; DhβE, Dihydro-β-erythroidine, HDAC, histone deacetylase; H3K9, histone H3K9; H3K14, histone H3K14; MLA, methyllycaconitine; NFAT, nuclear factor of activated T cells; NFATc2, nuclear factor of activated T cells 2; P, phosphorylation.

Such a dose-dependent effect of nicotine/smoking might be explained by the selective stimulation of the different types of nAChR with calcium influx (Figure 2) [9,17,24]. The neuronal nAChR subunits are currently classified into the α-type and the β-type in a neuron. A limited number of αβ or α-only subunit combinations have been found to form functional pentameric ion channels. The most common and widely distributed combinations of subunits are α7 and α4β2 [33]. MSCs and articular chondrocytes express both α7-nAChR and α4β2-nAChR [9,15,17,24]. Stimulation of nAChR accompanied by low-functional programming of IGF-I subsequently promoted chondrocyte proliferation through the phosphorylation of the ERK/MAPK pathway [17]. Moreover, evidence showed that SOX9 expression is regulated by the phosphatidylinositol-3-kinase/protein kinase B pathway and the Ca2+/calcineurin/NFATc2 signaling pathway [9]. However, the precise mechanism of such a selective stimulation of nAChR after nicotine exposure is not yet completely understood.

Nicotine also has a dose-dependent effect on the quality of the articular cartilage in adult offspring, OA susceptibility of offspring, and severity of MIA-induced OA. In animals, Tie et al. reported that PNE induced impaired articular cartilage quality in adult offspring and increased OA susceptibility of offspring [19,28]. However, the studies by Liu and Teng suggested that nicotine suppressed MIA-induced OA in animals with a dose of 0.5 or 1.0 mg/kg/d.

This preclinical evidence suggest that a dosage threshold might exist at which the negative effects of nicotine begin and thus may be avoided. The dose of nicotine exposure (0.5 and 1 mg/kg/d) for the available mice study is equivalent to 2 and 4 cigarettes/day for a 60 kg human. The arterial nicotine concentration was reported ranging from 0.12 to 0.6 μM after smoking one cigarette [34]; therefore, the dose of nicotine exposure as 0.5 and 1 mg/kg/d in animals may be equivalent less than 1 μM nicotine. Else, the epidemiologic evidence in humans highlighted the negative effects of cigarette smoking on knee osteochondral repair and joint degeneration, surfacing as cartilage loss and defect development primarily in individuals with an OA family history [13]. A recent meta-analysis reported that smokers had less radiographic OA than non-smokers [14]; however, men who smoke might die from other smoking-related diseases such as esophageal or lung cancer before OA may develop.

PNE induced an imperfect cartilage quality in offspring. Xie et al. showed that PNE intergenerationally programs impaired articular cartilage through maternal lineage [28]. A previous epidemiologic investigation based on 173,687 malformed cases and 11.7 million controls revealed that maternal smoking was associated with birth defects, including congenital osteochondral defects [35]. Additionally, Tie et al. demonstrated PNE induced fetal overexposure to maternal glucocorticoids, repressing the IGF-I expression in the fetus and inducing the intrauterine growth retardation [17]. Xie et al. also reported that corticosterone suppressed the expression of mRNAs related to SOX9, Col2a1, aggrecan, and TGF-β pathway components [28]. The acetylation modifications H3K9ac in TGF-βR1 and Col2a1 promoter were significantly reduced with 1250 nM corticosterone, and its inhibitor reversed such effects, however, 100 μM nicotine did not alter the acetylation at H3K9. These data indicate that corticosterone might mediate the intergenerational transmission of imperfect cartilage by PNE through the alteration of histone acetylation.

Finally, nicotine negatively influenced MSC-based osteochondral repair, perhaps through a decreased expression of Col2a1 following suppressing of SOX9 in a rat model [9]. This report is in good agreement with the known inhibitory effects of nicotine on MSC chondrogenesis in vitro [9,22,23] since MSC is a key cell type involved in spontaneous and therapeutic osteochondral repair [36,37].

This systematic review holds some limitations. A source of selection bias is inherent in the study inclusion criteria related to the use of only English language studies. Other sources of potential selection, performance, and detection bias include the analysis of only the knee joint in the animal experiments (versus other musculoskeletal regions and tissue types, which likely are equally affected by the systemic effects of smoking). As a result of these differences, it becomes a challenge to generalize and draw conclusions between the various studies with several different variables. No clinical study was included, which is outside of the scope of this work, focusing solely on in vitro and translational data.

For the future, the key question remains whether a threshold exists for the specific pathologies at which the negative effects of nicotine begin, and therefore, may be avoided, necessitating more specific investigations into dose-dependent clinical effects of smoking on cartilage quality in offspring, OA susceptibility and progression and osteochondral repair.

## Supplementary Materials

The following are available online at https://www.mdpi.com/2077-0383/8/10/1699/s1, Table S1: In vitro studies of effects of nicotine on chondrogenesis and osteogenesis of medicinal signaling cells, Table S2: In vitro studies of effects of smokers tobacco extract (STE) on osteoblast differentiation, Table S3: In vitro studies of effects of nicotine on articular chondrocytes, Table S4: Animal study of effect of nicotine on cartilage defect repair and OA, Table S5: Effects of prenatal nicotine exposure of animals knee cartilage development and OA susceptibility in offspring.
