# Supplementary material for: Association of Nicotine with Osteochondrogenesis and Osteoarthritis Development: The State of the Art of Preclinical Research
Source: J Clin Med. 2019 Oct 16;8(10):1699. doi: 10.3390/jcm8101699 (PMC6832988; doi:10.3390/jcm8101699)
Supplement: Supplementary file 1 [file jcm-08-01699-s001.pdf]

Supplementary materials

Association of Nicotine with Osteochondrogenesis and Osteoarthritis Development: The State of the Art of Preclinical Research

Xiaoyu Cai [1], Liang Gao [1], Magali Cucchiarini [1] and Henning Madry [1,2,*]

[1] Center of Experimental Orthopaedics, Saarland University Medical Center and Saarland University, 66421 Homburg/Saar, Germany; xiaoyucai0418@gmail.com (X.C.), liang.gao@uni-saarland.de (L.G.), mmcucchiarini@hotmail.com (M.C.)

[2] Department of Orthopaedic Surgery, Saarland University Medical Center and Saarland University, 66421 Homburg/Saar, Germany

* Correspondence: henning.madry@uks.eu; Tel.: +49-06841-1624569

Table S1. In vitro studies of effects of nicotine on chondrogenesis and osteogenesis of medicinal signaling cells.

Nicotine dose	Cell type and source	Treatment groups	Follow-up	Measures	Outcomes	Conclusions	Refs.
0, 0.1, 1 and 10 μM	Human BMSCs from the iliac crest	1. Nicotine group: 0.1, 1, and 10 μM nicotine<br><br>2. Control group: DMEM with 10% (V/V) FBS with 50 lg/ml ascorbate-2-phosphate	21 days	CKK-8 assay: cell proliferation<br><br>Alcian blue staining: proteoglycan synthesis<br><br>DMMB assay: release of sGAG<br><br>PCR: expression of aggrecan, , type-I, -II, and -X collagen, and aggrecan mRNAs<br><br>ELISA: expression of type-II collagen protein	1. Cell proliferation: significantly increased with 1 μM nicotine at day 4, 7, and 14; significantly inhibited with 10 μM nicotine at day 7 and 14<br><br>2. GAG content: significantly reduced with 10 μM nicotine at day 14<br><br>3. Expression of type-II collagen mRNA: significantly up-regulated with 0.1 and 1 μM nicotine at day 7, 14, and 21<br><br>4. Expression of mRNA aggrecan: significantly reduced with 10 μM nicotine at day 14<br><br>5. Expression of fibroblastic marker genes (type-I collagen)-related mRNA: significantly down-regulated with 1 and 10 μM nicotine at day 7; significantly down-regulated with 0.1, 1, and 10 μM nicotine at day 14 and 21<br><br>6. Expression of hypertrophic marker genes (type-X collagen)-related mRNA: significantly down-regulated with 0.1, 1, and 10 μM nicotine at day 14 and 21<br><br>7. Expression of type-II collagen protein: increased singnificantly with 0.1 and 1 μM nicotine at day 7 (103.3 ± 14.6, 118.7 ± 12.5, P < 0.050), day 14 (123.8 ± 15.5, 118.7 ± 12.5, P < 0.050) , and day 21 (147.5 ± 16.3, 171.2 ± 18.4, P < 0.050)	1. Nicotine has a dose-dependent effect on expression of aggrecan, type-I, -II, and -X collagen in humam BMSCs.	[16]

25, 50, and 100 μM	Rat BM-MSCs from femur	1. Nicotine group: 25, 50, and 100 μM<br><br>2. Control group: DMEM with 10% (V/V) FBS	28 days	MTT assay: cell viability<br><br>Alcian blue and Safranin O staining: GAG quantification<br><br>PCR: expression of aggrecan, Col2a1 and IGF-I mNRAs	1. GAG extent: Alcian blue-stained area significantly reduced to 85%, 49% , and 5% with 25, 50 , and 100 μM after day 28, respectively; Safranin O-stained area significantly reduced to 91%, 72%, and 31% with 25, 50, and 100 μM after day 28, respectively<br><br>2. Expression of aggrecan, Col2a1, and IGF-I mRNAs: significantly decreased after continuous nicotine exposure after day 28	1. Nicotine suppressed chondrogenic differentiation potential of BM-MSCs with possible mechanism of decreasing the expression of aggrecan, Col2a1, and IGF-I mRNAs.	[1]
0.1, 1, 10 and 100 μM	Rat BM-MSCs from the tibias and femurs	1. Nicotine group: 0.1, 1, 10 and 100 μM<br><br>2. MLA group: 10 μM MLA and Si-NFATc2 for 0.5 h and then 100 μM nicotine<br><br>3. Control group: DMEM/F12 with 10% FBS	24 h	Calcineurin phosphatase activity assay: CaN activity<br><br>PCR and western blotting: expression of α7-nAChR, SOX9, and NFATc2 mRNAs and protein<br><br>ChIP assay: binding of NFATc2, HDAC1, H3K9ac, and H3K14ac on the SOX9 promoter; binding of SOX9 on Col2a1<br><br>Immunoprecipitation: interaction of NFATc2 and HDAC	1. CaN activity: significantly incresed with 0.1, 1, 10 and 100 μM nicotine at 0.5 h<br><br>2. Expression of SOX9 mRNA and protein: significantly decreased with 1, 10, and 100 μM nicotine at 24 h<br><br>3. Expression of NFATc2 mRNA and protein: nulceic NFATc2 protein significantly increased with 0.1, 1, 10 and 100 μM nicotine at 24 h; cytoplasm phosphorylated NFATc2 significantly reduced with 0.1, 1, 10 and 100 μM nicotine at 24 h<br><br>4. Binding of NFATc2 and HDAC1 on SOX9 promoter: significantly increased with 1, 10, and 100 μM nicotine at 24 h<br><br>5. H3K9ac and H3K14ac levels on SOX9 promoter: significantly reduced with 0.1, 1, 10 and 100 μM nicotine at 24-h treatment of 10 μM MLA or si-NFATc2, H3K9ac and H3K14ac levels significantly rescued<br><br>6. Binding of SOX9 and Col2a1 enhancer: significantly decreased with 0.1, 1, 10 and 100 μM nicotine at 24 h; treatment of 10 μM MLA or si-NFATc2, the binding of SOX9 and Col2a1 significantly rescued	1. Nicotine induced intracellular calcium in a concentration-dependent manner through α7-nAChR.<br><br>2. Nicotine suppressed SOX9 by activating the Ca2+/calcineurin/NFATc2 signaling pathway through α7-nAChR.<br><br>3. Nicotine decreased the histone acetylation on the promoter of SOX9.<br><br>4. NFATc2 could bind to SOX9 promoter and recruit HDAC1, then reduce H3K9ac and H3K14ac levels in the SOX9 promoter and decrease the expression of SOX9.	[10]

0, 0.5, 1, 5, and 10% (V/V%) of CSE; (3 Marlboro red cigarettes smoked for 15 mins)	Human AD-MSCs from lipoaspirates	1. Nicotine group: 0.5, 1, 5 and 10% CSE<br><br>2. Control group: DMEM with 10% (V/V) FBS	21 days	MTT assay: cell viability and metabolic activity<br><br>Scratch wound assay: cell migration<br><br>Antibody arrays: secretion profile<br><br>Alizarin Red S staining: calcium deposition<br><br>PCR: BGLAP, RUNX2, SPP1, ACAN, Col2a1, and SOX9 expression	1. Cell viability and metabolic activity: significantly impaired with 5% and 10% CSE at 48h<br><br>2. Cell migration: continual migration with 0.5% and 1% CSE; limited migration with 5% CSE; cells scarcely survived with 10% CSE at 48h<br><br>3. Expression of BGLAP mRNA: no expression in nicotine group at day 3; similar expression with control group after day 7; doubled expression with 0.5% CSE after day 14<br><br>4. Secretion of IL-6 and IL-8: significantly decreased with 0.5% CSE at 48h<br><br>5. Expression of RUNX2 mRNAs: significantly higher with 0.5% CSE at day 3, 7, and 14<br><br>6. Expression of ACAN and SOX9 mRNAs: significantly higher with 0.5% CSE at day 7 compared with day 14 and 21	1. CSE exposure with concentrations higher than 5% drastically impaired the cell viability and migration.<br><br>2. While some recovery is possible with low dose CSE exposure, lasting effects exist within the AD-MSCs for cellular signaling with impaired osteogenic-and chondrogenic lineages.	[12]
E-cigarette smoke extract containing 18 mg/ml nicotine	Human BM-MSCs	1. E-cigarette smoking group<br><br>2. Control group	10 days	Oosteogenic markers<br><br>Reactive oxygen species<br><br>Intercellular communication by fluorescence recovery after photo-bleaching (FRAP)	1. Significantly decreased collagen I and Runx2 expression<br><br>2. Signfiicantly altered cellular morhoplogy with less mineralization<br><br>3. More reactive oxygen species generated by smoke extract	E-cigarette smoke extracts attenuate MSC differentiation, enhances reactive oxygen species production and inhibits cell-cell communication .	[7]
5 µM	Human WJ-MSCs from umbilical cord	1. Nicotine group: 5 µM<br><br>2. Control group: αMEM with 10% (V/V) FBS	28 days	MTT assay: cell proliferation and viability<br><br>Haematoxylin-Erythrosine-Safran staining: cell morphology<br><br>Sirius red and Alcian blue staining: collagen	1. Cell proliferation: significantly decreased after 3-day with 5 µM nicotine<br><br>2. Proteoglycan synthesis: significantly decreased with 5 µM after day 28<br><br>3. Expression of SOX9, Col2a1, and aggrecan mRNAs: significantly down-regulated with 5 µM nicotine after day 28<br><br>4. Expression of α7-nAChR mRNA: expressed and activated with 5 µM nicotine after day 28	1. Nicotine impaired the cellular proliferation and chondrogenic differentiation.<br><br>2. Nicotine had no significant effects on the cell viability of WJ-MSCs.	[14]

				and proteoglycan synthesis<br><br>PCR : expression of SOX9, Col2a1, aggrecan, and α7-nAChR mRNAs<br><br>Calcium assay: function of nAChR			
0, 0.5, and 1.0 μM	Human BM-MSCs (Cat #7500) and PDLSCs	1. Nicotine group: 0.5 and 1 μM nicotine<br><br>2. Control group: DMEM with 10% (V/V) FBS	10 days	MTT assay: cell proliferation<br><br>Scratch wound assay: cell migration<br><br>Alizarin Red S staining: calcium depositon<br><br>Burstone's staining: alkaline phosphatase<br><br>PCR: expression of PTK2, RUNX2	1. Cell proliferation: significantly lower with 1 μM nicotine at day 5<br><br>2. Cell number: over 2-fold decrease with 1 μM nicotine at day 5<br><br>3. Cell migration: movement distance significantly shorter in nicotine group (BM-MSCs: 22.61 ± 3.98 μm, P = 0.020; PDLSCs: 3.50 ± 0.86 μm, P = 0.008)<br>with 1 μM nicotine at day 5; Migration speed: significantly slower in nicotine group (BM-MSCs, 3.14 ± 0.55 nm/s, P = 0.024; PDLSCs, 0.49 ± 0.12 nm/s, P = 0.008) with 1 μM nicotine at day 5<br><br>4. Osteogenesis related miRNAs of PDLSCs: significantly up-regulated (hsa-miR-29b: 0.5 M nicotine, + 1.194-fold; 1 M nicotine, + 1.217-fold; hsa-miR-30d: 0.5 M nicotine, + 3.823-fold; 1 M nicotine, + 46.225-fold; hsa-miR-137: 0.5 M nicotine, + 2.412-fold; 1 M nicotine, + 3.156-fold; hsa-miR-424: 0.5 M nicotine, + 1.144-fold; 1 M nicotine, + 1.217-fold; and hsa-miR-1274a: 0.5 M nicotine, + 1.119-fold; 1 M nicotine, + 1.207-fold)<br><br>5. Expression of PTK2 and RUNX2 mRNAs: significantly down-regulated (BM-MSCs: - 3.0- and - 9.1-fold, P < 0.010; PDLSCs: - 2.1- and - 2.1-fold, P < 0.010) with 1.0 μM nicotine at day 3<br><br>6. Expression of ALP, BGLAP, Col1a1, and Col1a2 mRNAs: significantly down-regulated (-2- to -5.3-fold) with 1 μM nicotine at day 3	1. Proliferation, migration, and osteogenic differentiation of human MSC and PDLSCs were inhibited with nicotine.<br><br>2. miRNAs were significantly up-regulated with 1 μM with the nicotine dose-dependent changes from 0.5 to 1 μM nicotine.<br><br>3. miRNAs are a key regulator in the nicotine-associated functional changes.	[6]

No concentration of nicotine	PDLSCs from cigarette smokers	1. Smokers group: 3 cigarette smokers (33 ± 7.21 years old) with smoking hisotory during the past 30 days<br><br>2. Non-smokers group: 3 age matched non-smokers	14 days	MTT assay: cell proliferation<br><br>Scratch wound assay: cell migration<br><br>Burstone's staining: alkaline phosphatase<br><br>Alizarin Red S staining: calcium depositon<br><br>Alcian blue 8GX staining: acidic polysaccharide<br><br>PCR: microRNA expression	1. Cell proliferation: significantly lower in smokers group by 2.53-fold at day 5 and 2.88-fold at day 7<br><br>2. Cell migration: significantly decreased in smokers group at 12 and 24 h<br><br>3. miRNAs expression : two significantly up-regulated (hsa-miR-1305: + 22.08-fold, hsa-miR-18b: + 15.56-fold); one significantly down-regulated (hsa-miR-3198: - 42.98-fold)	1. Cigarette smoke extract inhibits osteogenic differentiation of human osteoprogenitor cells, and nicotine also reduces osteogenic differentiation of PDLSCs.<br><br>2. The proliferation, migration, and differentiation potential of PDLSCs into osteoblasts and chondrocytes from smokers were inhibited.<br><br>3. miRNAs might play an important role in the deteriorative effects on stem cells by cigarette smoke.	[5]

Abbreviations: ACAN, aggrecan; AD-MSCs, adipose-derived mesenchymal stem cells; ALP, alkaline phosphatase; αMEM, alpha modified Eagle's medium; BGLAP, bone gamma-carboxyglutamic acid-containing protein; BM-MSCs, bone marrow-derived mesenchymal stem cells; BMSCs, bone marrow stromal cells; CCK-8 assay, cell counting kit-8 assay; ChIP assay, chromatin immunoprecipitation assay; CSE, Cigarette smoke extract; Col1a1, α1 chain of type-I collagen; Col1a2, α2 chain of type-I collagen; Col2a1, α1 chain of type-II collagen; DMEM, Dulbecco's modified Eagle's medium; DMMB assay: Dimethylmethylene Blue assay; FBS, fetal bovine serum; GAG, glycosaminoglycan; HDAC, histone deacetylase; WJ-MSCs, Wharton's jelly-derived mesenchymal stem cells; IGF-I: insulin-like growth factor-I; IL-1, interleukin-1; IL-6, interleukin-6; IL-8, interleukin-8; miRNA, microRNA; MLA, methyllycaconitine; MTT assay, Methyl thiazolyl tetrazolium assay; α7-nAChR, α7-nicotinic acetylcholine receptor; NFATc2, nuclear factor of activated T cells 2; PCR, polymerase chain reaction; PDLSCs, periodontal ligament-derived stem cells; PTK2, protein tyrosine kinase 2; RUNX2, Runt-related transcription factor 2; sGAG, sulfated glycosaminoglycan; Si-NFATc2, NFATc2 siRNA; SOX9, SRY-type high mobility group box 9; SPP1, secreted phosphoprotein 1.

Table S2. In vitro studies of effects of smokeless tobacco extract (STE) on osteoblast differentiation.

**Nicotine dose**	**Cell type and source**	**Treatment groups**	**Follow-up**	**Measures**	**Outcomes**	**Conclusions**	**Refs.**
STE from 75 g smokless tobacco	Osteoblasts from chick embryo clavarias	Osteoblasts treated with different cenentrations of smokeless tobacco extract	25 days	Alkaline phosphatase activity<br><br>Collagen protein content	1. Alkaline phosphatase activity of osteoblasts treated with smokeless tobacco extract was significantly decreased.<br><br>2. [^{3}H]hydroxyproline and [^{3}H]proline content in the cell layer of osteoblasts were decreased 50% and 29%, respectively.	Extract of smokeless tobacco irreversibly inhibts osteoblastic differentation with decreased bone collagen synstehsis.	3
STE from 300 g smokeless tobacco; dilated to 10^2, 10^3, 10^4, 10^5, and 10^6	Osteoblasts from chick embryo clavarias	Osteobalsts without or with treatment of diluated STE and IGF-I	7 days	Cell proliferation<br><br>Protein content and alkaline phosphatase activity<br><br>Bone deposition and bone nodule mineralization	1. Cell proliferation was significantly stimulated by 102–104 diluted smokeless tobacco extract.<br><br>2. Alkaline phosphatase activity was significantly increased by 102–104 diluted smokeless tobacco extract but decreased by 106 diluted smokeless tobacco extract.<br><br>3. Low dilution (103 and 104) significantly increased bone nodule formation and inhibited their mineralization.<br><br>4. High dilution (105 and 106) significantly decreased bone nodule formation, but increased their mineralization.<br><br>5. Heat and acid treatment of STE significantly reduced its beneficial effect on cell proliferation.	STE may contain a therapeutic peptide to stimulate osteoblast proliferation, differentiation and metabolism, comparable to the effects of IGF-1.	2

Abbreviations: IGF-I, insulin-like growth factor 1 ; STE, smokeless tobacco extract.

Table S3. In vitro studies of effects of nicotine on articular chondrocytes.

Nicotine dose	Cell type and source	Treatment groups	Follow-up	Measures	Outcomes	Conclusions	Refs.
0, 0.15, 0.3 and 0.6 μM	Human normal and OA articular chondrocytes from knee joints	1. Normal chondrocytes<br>2. OA chondrocytes<br><br>Both group were treated without or with nicotine (0, 0.15, 0.3, and 0.6 μM)	7 days	MTT assay: cell proliferation<br><br>PCR: expression of type-II collagen and aggrecan mRNAs<br><br>ELISA: expression of type-II collagen and aggrecan protein	1. Cell proliferation: significantly increased with nicotine in both groups at day 1, 4, and 7 in a concentration- and time-dependent manner<br><br>2. Expression of type-II collagen mRNA: significantly increased (1.7- and 2.5-fold in normal chondrocytes, 1.3- and 1.5-fold in the OA chondrocytes with 0.15 and 0.3 μM nicotine at day 4; 3.6- and 4.4-fold in normal chondrocytes, 1.6- and 2.2-fold in the OA chondrocytes with 0.15 and 0.3 μM nicotine at day 7) compared ith control in both groups<br><br>3. Expression of type-II collagen and aggrecan mRNAs: significantly less in OA chondrocytes compared with in the normal chondrocytes<br><br>4. Expression of type-II collagen and aggrecan protein: significantly increased with 0.15, 0.3, and 0.6 μM both at day 4 and 7	Nicotine promotes the proliferation of human normal and OA articular chondrocytes and enhances the expression of type-II collagen.	[15]
0, 0.4, 2 and 10 μM	Fetal rat articular chondrocytes from distal femoral and proximal tibial cartilage	1. Nicotine group: 0.4, 2, 10 μM<br><br>2. DHβE group: 1 μM DHβE for 0.5 h and then 10 μM nicotine<br><br>3. Control group: DMEM/F12 with 10% FBS	10 days	PCR: expression of nAchR, Col2a1, aggrecan, MMP-3, MMP-13, ADAMTS-4/5, IGF-I, IRS1, AKT1, and SOX9 mRNAs<br><br>Western blotting: expression of Col2a1, aggrecan, MMP-3, MMP-13, ADAMTS-4/5, IGF-I, IRS1, AKT1/2, and SOX9 protein<br><br>DMMB assay: release of GAG	1. Expression of α4β2-nAchR mRNA: expressed in the fetal articular chondrocytes<br><br>2. Expression of Col2a1 mRNA: significantly decreased with 0.4 , 2, and 10 μM nicotine<br><br>3. Expression of Col2a1 protein: significantly decreased with 0.4, 2, and 10 μM nicotine<br><br>4. Expression of aggrecan and SOX9 mRNAs: significantly decreased with 0.4, 2, and 10 μM nicotine<br><br>5. Release of GAG: significantly decreased with 0.4, 2, and 10 μM nicotine<br><br>6. Expression of catabolic genes (MMP-3, MMP-13, and ADAMTS-4/5) related mRNAs: significantly increased with 0.4, 2, and 10 μM nicotine<br><br>7. Expression of molecules in IGF-I signaling pathway (IGF-I, IRS1, AKT1) related mRNAs: significantly	1. Nicotine dose-dependently suppressed the Col2a1, aggrecan, IGF-I signaling pathway and SOX9 expression.<br><br>2. α4β2-nAchR mediated the effects of nicotine on fetal rat articular chondrocytes.	[11]

					decreased with 0.4, 2, and 10 μM nicotine<br><br>8. Expression of molecules in IGF-I signaling pathway (IGF-I, IRS1, and AKT1) related protein: significantly decreased with 0.4, 2, and 10 μM nicotine<br><br>9. Expression of SOX9 protein: significantly decreased with 2 and 10 μM nicotine<br><br>10. DHβE: rescued the expression of Col2a1, IGF-I, IRS1, AKT1, and SOX9 related mRNAs and protein; expression of aggrecan mRNA; and release of GAG		
0.1, 1, 10 and 100 μM	Fetal rat articular chondrocytes from knee joint	1. Nicotine group: 0.1, 1, 10 and 100 μM<br><br>2. Corticosterone group: 125, 250, 500 and 1,250 nM<br><br>3. TGF-βR1 inhibitor group: LY2157299<br><br>4. TGF-βR1 inhibitor and corticosterone group: LY2157299 30 min then corticosterone for 48 h<br><br>5. α7-nAchR inhibitor group: 10 μM MLA for 0.5 h followed by 100 μM nicotine<br><br>6. Corticosterone receptor inhibitor group: 5 μM MIF fo 0.5 h followed by 1250 nM corticosterone	48 h	MTS assay: cell viability<br><br>ChIP assay: histone acetylation<br><br>PCR: SOX9, Col2a1, ACAN, TGF-β, TGF-βR1, Smad2, and Smad3 expression	1. Expression of SOX9 mRNA: significantly inhibited with 1, 10, and 100 μM nicotine at 48 h<br><br>2 Expression of Col2a1 mRNA: significantly inhibited with 0.1, 1, and 100 μM nicotine at 48 h<br><br>3. Expression of ACAN mRNA: significantly inhibited with 0.1, 1, 10, and 100 μM nicotine at 48 h<br><br>4. Expression of TGF-β and Smad2 mRNAs: significantly inhibited with 250 nM, 500, and 1250 nM corticosterone at 48 h<br><br>5. Expression of SOX9 and ACAN mRNAs: significantly inhibited with 125 nM, 250, 500 and 1,250 nM corticosterone at 48h<br><br>6. Expression of TGF-βR1 mRNAs: significantly inhibited with 250, 500nM, and 1250 nM corticosterone at 48h<br><br>7. Expression of Smad3 mRNA: significantly inhibited with 125, 250 nM, 500, and 1250 nM corticosterone at 48h<br><br>8. Expression of Col2a1 mRNA: significantly inhibited with 250 nM, 500, and 1250 nM corticosterone at 48h<br><br>9. Expression of Col2A1 and ACAN mRNAs: significantly reduced in LY2157299 group and LY2157299 + corticosterone group	1. Nicotine could not inhibit the TGF-β signaling pathway directly in rat fetal chondrocyte. Suppression of TGF-β axis induced by PNE might be mediated by corticosterone .<br><br>2. Corticosterone induced the hypoacetylation at H3K9 of TGF-βR1 and Col2a1 gene, but nicotine did not alter the acetylation at H3K9.	[13]

					10. Acetylation modifications of H3K9ac in TGF-βR1 and Col2a1 promoter: significantly reduced with 1250 nM corticosterone ; 5 µM of MIF reversed the effect of 1250 nM corticosterone		
10 µM	SD rat articular chondrocytes from femoral head caps	1. MIA group: 10 µM for 4 h<br><br>2. MIA + nicotine group: 10 µM MIA 0.5 h + 10 µM nicotine for 0.5 h<br><br>3. MIA + nicotine + MLA group: 10 µM<br>MIA for 0.5 h + 10 µM nicotine for 0.5 h + MLA 10 nM for 0.5 h<br><br>4. Control group: DMEM/F12 with 10% (V/V) FCS	4 h	Western blotting: expression of α7-nAchR, p38, ERK1/2, JNK protein	1. Expression of α7-nAchR protein: α7-nAchR subunit protein expressed<br><br>2. Phosphorylation of p38, ERK1/2 and JNK induced by MIA: significantly induced by 10 µM MIA after 1 h; peak levels occurred after 2 h; significantly higher in MIA group, MIA + nicotine group, and MIA + nicotine + MLA group than control group (significantly lower in MIA + nicotine group compared with MIA group; significantly lower in MIA + nicotine group compared with MIA + nicotine + MLA group<br><br>3. Phosphorylation of p38, ERK1/2 and JNK induced by IL-1β: significantly induced by 10 ng/ml IL-1β after 0.5 h; peak levels occurred after 0.5 h; significantly higher in IL-1β group, IL-1β + nicotine group, and IL-1β + nicotine +MLA group than control group; significantly lower in IL-1β + nicotine group compared with IL-1β group; significantly lower in IL-1β + nicotine group compared with IL-1β + nicotine + MLA group<br><br>4. Phosphorylation of NF-κB p65 induced by MIA: significantly induced by 10 µM MIA after 1 h; significantly higher in MIA group, MIA + nicotine group, and MIA + nicotine +MLA group than control group; significantly lower in MIA + nicotine group compared with MIA group; significantly lower in MIA + nicotine group compared with MIA + nicotine +MLA group<br><br>5. Phosphorylation of NF-κB p65 induced by IL-1β: significantly induced by 10 ng/ml IL-1β from 0.25 to 2 h; significantly higher in IL-1β group, IL-1β + nicotine group, and IL-1β + nicotine +MLA group than control group; significantly lower in IL-1β + nicotine group compared with IL-1β group; significantly lower in IL-1β + nicotine group compared with IL-1β + nicotine +MLA group	1. The α7-nAchR subunit was expressed in chondrocytes.<br><br>2. Nicotine inhibited MIA- or IL1β-induced chondrocyte activation via α7-nAChRs in vitro and decreased the resulting phosphorylation of p38, ERK1/2 and JNK MAPKs and NF-κB p65.	[4]

Abbreviations: ACAN, aggrecan; ADAMTS-4/5, a disintegrin and metalloproteinase with thrombospondin motifs 4/5; AKT1/2, serine–threonine protein kinase 1/2; α4β2-nAchR, α4β2-nicotine acetylcholine receptor; Col2a1, α1 chain of type-II collagen; ChIP assay, chromatin immunoprecipitation assay; DHβE, dihydro-β-erythroidine; DMEM, Dulbecco's modified Eagle's medium; DMMB assay: Dimethylmethylene Blue assay; ELISA, enzyme-linked immunosorbent assay; ERK1/2, extracellular signal-regulated kinase 1/2; FBS, fetal bovine serum; FCS, fetal calf serum; F12, Ham's F 12 nutrient medium; GAG, glycosaminoglycan; IL-1β, Interleukin-1β; IRS-1, Insulin receptor substrate 1; JNK, c-JUN N-terminal kinase; LY2157299, TGF-βR1 inhibitor; MIA, monosodium iodoacetate; MIF, mifepristone; MLA, methyllycaconitine; MMP-3, matrix metalloproteinase 3; MMP-13, matrix metalloproteinase 13; MTS assay: 3-(4,5-dimethylthiazol-2-yl)-5-(3-carboxymethoxyphenyl)-2-(4-sulfophenyl)-2H-tetrazolium) assay; MTT assay, Methyl thiazolyl tetrazolium assay; NF-κB, nuclear factor-kappa B; PCR, polymerase chain reaction; SD rat, Sprague-Dawley rat; Smad2/3, SMAD Family Member 2/3; SOX9, SRY-type high mobility group box 9; TGF-β, transforming growth factor-β; TGF-βR1, transforming growth factor-β receptor 1.

Table S4. Animal study of effect of nicotine on cartilage defect repair and OA.

Nicotine dose	Animal model; defect details	Administration	Treatment groups	Follow-up	Measures	Outcomes	Conclusions	Refs.
2 mg/kg/d	Rat osteochondral defect in the trochlear groove (2-mm diameter, 3-mm depth). All defects received BM-MSCs (2 × 10^6 cells/ml) suspended in 1.25 % alginate intraoperatively	Subcutaneous injection: 1 mg/kg twice per day for 10 days in nicotine group, same volume of saline in control group	1. Nicotine group: 2 mg/kg/d 2. Control group: same valume of saline	12 weeks	ICRS macroscopic and Wakitani score (Safranin O/Fast green staining): cartilage repair Immunohistological staining: Col2a1 and SOX9 expression PCR: Col2a1, aggrecan, and SOX9 expression	1. ICRS macroscopic scores: significantly lower in nicotine group at week 12 2. Wakitani histological scores: significantly higher in nicotine group at week 12 3. Expression of Col2a1, aggrecan and SOX9 mRNAs and protein: significantly decreased in nicotine group at week 12	1. Nicotine impaired cell morphology of the regenerated tissue and synthesis of cartilage matrix. 2. Nicotine decreased the expression of Col2a1 by suppressing SOX9 and negatively influenced the cartilage defect repair.	[10]
1 mg/kg/d	Male rats treated with nicotine, MLA, MIA	Intra-articular injection: 1 mg of MIA in 50 µl of sterile physiologic saline solution once a week for 4 weeks in MIA group; same volume of saline in control group intraperitoneal	1. MIA group: 1 mg/week for 4 weeks 2. Nicotine +MIA group: 1 mg/week MIA for 4 weeks and 1 mg/kg/d nicotine for 5 weeks 3. MIA +	6 weeks	Macroscopic scores: joint destruction Mankin score (H&E and toluidine blue staining): joint destruction	Macroscopic scores and Mankin score: significantly higher in MIA group, MIA + nicotine group, and MIA + nicotine +MLA group than control group at week 6; significantly lower in MIA + nicotine group compared with MIA group at week 6; significantly lower in MIA + nicotine group compared with MIA + nicotine +MLA group at week 6	Nicotine acting via α7-nAChRs prevents MIA-induced OA in rats.	[4]

		injection: 1 mg/kg/d of nicotine once for 5 weeks; 1 mg/kg/d of MLA 30 min before nicotine administration	nicotine + MLA group: 1 mg/week MIA for 4 weeks, 1 mg/kg/d nicotine, and 1 mg/kg/d MLA for 5 weeks<br><br>4. Control group: same volume of saline for 5 weeks					
0.5 or 1 mg/kg/d	Wild-type male mice treated with nicotine, MLA, MIA	Intraperitoneal injection: 0.5 or 1 mg/kg of nicotine 30 min before each MIA injection once per day for 4 week in nicotine treatment group; 1 mg/kg of MLA 30 min before nicotine injection for the nicotine antagonism treatment; equal volume of saline in the control group;<br><br>intra-articular injection: 0.1 mg of MIA in 10 µl of sterile saline at day 7 in MIA group, MIA + nicotine group, and MIA + nicotine + MLA group	1. MIA group: 0.1 mg<br><br>2. MIA + nicotine group: 0.1 mg MIA and 0.5 or 1 mg/kg/d nicotine for 4 weeks<br><br>3. MIA + nicotine + MLA group: 0.1 mg MIA, 1 mg/kg/d nicotine, and 1 mg/kg/d MLA for 4 weeks<br><br>4. Control group: same volume of saline for 4 weeks	4 weeks	Behavioral test (von Frey hair): mechanical sensitivity<br><br>Aggrecan loss scores and cartilage degeneration scores: cartilage degradation	1. Mechanical sensitivity: significantly lower in MIA group, MIA + nicotine 0.5 mg/kg group, and MIA + nicotine 1 mg/kg + MLA group compared with control group during day 10-28; significantly lower in MIA + nicotine 1 mg/kg group compared with control group during day 10-28; significantly higher in MIA + nicotine 0.5 mg/kg group and MIA + nicotine 1 mg/kg group compared with MIA group during day 10-28; significantly higher in MIA + nicotine 1 mg/kg group compared with MIA + nicotine 1 mg/kg + MLA group during day 10-28<br><br>2. Aggrecan loss scores, cartilage degeneration scores, and protein expression of MMP-9 : significantly increased in MIA group and MIA + nicotine 1 mg/kg + MLA group versus the control group; significantly lower in MIA + nicotine 0.5 mg/kg group and MIA + nicotine 1 mg/kg group versus MIA group; significantly higher in MIA + nicotine 1 mg/kg + MLA group versus MIA + nicotine 1 mg/kg group	1. Nicotine inhibited OA-induced mechanical allodynia in mice.<br><br>2. Nicotine suppressed MIA-induced OA by activating α7-nAChRs in mice.	[8]

Abbreviations: Col2a1, α1 chain of type-II collagen; ICRS, International Cartilage Repair Society; MIA, monosodium iodoacetate; MIF, mifepristone; MLA, methyllycaconitine; OA, osteoarthritis; PCR, polymerase chain reaction; SOX9, SRY-type high mobility group box 9.

Table S5. Effects of prenatal nicotine exposure of animals knee cartilage development and OA susceptibility in offspring.

Nicotine dose	Animal model; defect details	Administration	Treatment groups	Follow-up	Measures	Outcomes	Conclusions	Refs.
2 mg/kg/d	Prenatal rat treated with nicotine; offspring without treatment	Subcutaneous injection: 1 mg/kg twice per day for 12 days for nicotine group, same volume of saline for control group	1. PNE group: 2 mg/kg/d<br><br>2. Control group: saline	30 weeks	H&E and Safranin-O/Fast green staining: cartilage quality<br><br>Immunohistochemical assay: expression of Col2a1, TGF-β, TGF-βR1, smad2, smad3 and SOX9 protein<br><br>PCR: expression of Col2a1, ACAN, TGF-β, TGF-βR1, smad2, smad3 and SOX9 mRNAs<br><br>ChIP assay: histone acetylation	1. Cartilage repair: significantly reduced in PNE-F1 group at both GD 20 and PW 12, and PNE-F2 group at PW 12<br><br>2. Expression of Col2a1 protein: significantly decreased in the PNE-F1 at both GD 20 and PW 12, and PNE-F2 group at PW 12<br><br>3. Expression of Col2a1 and ACAN mRNAs: significantly decreased in the PNE-F1 at both GD 20 and PW 12, and PNE-F2 group at PW 12<br><br>4. Expression of TGF-β, TGF-βR1, smad2, and SOX9 protein: significantly decreased in PNE-F1 at both GD 20 and PW 12, and PNE-F2 group at PW 12<br><br>5. Expression of TGF-β, TGF-βR1, smad2, smad3 and SOX9 mRNAs: significantly decreased in PNE-F1 at both GD 20 and PW 12, and PNE-F2 group at PW 12<br><br>6. Acetylation modification at H3K9 of TGF-β, TGF-βR1, SOX9, Col2a1, and ACAN gene promoters in PNE-F1 and PNE-F2: decreased significantly in PNE-F1 at both GD 20 and PW 12, and PNE-F2 group at PW 12	1. Effect of PNE on the articular cartilage of the female F1 generation lasted from fetus to adult.<br><br>2. PNE induced an imperfect cartilage quality for the first even second adult rat offspring by inhibiting histone acetylation of TGF-β and cartilage ECM promoters and down-regulating their expression.<br><br>3. Histone deacetylation at the site H3K9 of TGF-βR1 and Col2a1 promoters reserved in PNE-F2 may be mediated by the effect of CORT but not nicotine directly.	[13]

2 mg/kg/d	Prenatal rat treated with nicotine; offspring fed with a high-fat diet	Subcutaneous injection: 1 mg/kg twice per day for 10 days for nicotine group, same volume of saline for control group	1. PNE group: 2 mg/kg/d<br><br>2. Control group: same valume of saline<br><br>3. High-fat diet experiments: 88.0% corn flour, 11.5% lard, and 0.5% cholesterol	27 weeks	Tissue total cholesterol biochemical assay: cholesterol content of cartilage<br><br>ELISA: serum corticosterone and IGF-I concentrations<br><br>Biochemical assay: serum total cholesterol HDL-C, and LDL-C concentrations<br><br>Total cholesterol biochemical assay: cartilage total cholesterol<br><br>OARSI scores (Safranin-O/Fast green staining): cartilage quality<br><br>Immunohistochemical assay: protein Expression of Col2a1, IGF-I, PPARγ, LXR β, and ABCA1<br><br>PCR: mRNA expression of IGF-I, LXR β, ABCA1, and Col2a1	1. Expression of Col2a1 protein: significantly reduced in PNE group at PW 24<br><br>2. OARSI scores: significantly higher in PNE group at PW 24<br><br>3. Cholesterol content of cartilage: significantly increased in PNE group at PW 24<br><br>4. Serum corticosterone levels: significantly decreased in nicotine group at PW 24<br><br>5. Serum IGF-I, total cholesterol and LDL-C level: significantly increased in nicotine group at PW 24<br><br>6. Expression of IGF-I, LXR β, and ABCA1 protein: significantly decreased in nicotine group at both GD 20 and PW 24<br><br>7. Expression of IGF-I, LXR β, ABCA1, and Col2a1 mRNAs: significantly decreased in nicotine group at GD 20	1. Cholesterol deposited in the articular cartilage of female adult offspring with PNE might attribute to the reduced cartilage quality.<br><br>2. PNE suppressed IGF-I expression and down-regulated the expression of the cholesterol efflux pathway proteins.<br><br>3. PNE induced low articular cartilage quality in female adult offspring fed with a high-fat diet.	9
2 mg/kg/d	Prenatal rat treated with nicotine; offspring treated with strenuous running as method of OA induction (total of 30 km in 6 weeks induced OA after weaning PW 3	Subcutaneous injection: 1 mg/kg twice per day for 10 days for PNE group; same volume of	1. Running PNE group: nicotine 2 mg/kg/d during GD 10-20; running for 30 km within 6 weeks inducing OA<br><br>2. Running control group: saline 2 mg/kg/d during GD 10-20;	27 weeks	India ink staining: cartilage destruction<br><br>Mankin score: cartilage quality<br><br>PCR: expression of Col2a1, IGF-I, AKT1/2, and SOX9 mRNAs	1. Cartilage quality: HC thickness significantly decreased in running PNE group compared with running control group at PW 24; clacified cartilage thickness significantly increased in both running and non-running PNE group at PW 24<br><br>2. Mankin scores: significantly higher in running group than	1. PNE induces epigenetic modification in genes of IGF-I signaling or ECM genes, thereby inducing	11

	weeks in group both PNE and control group)	saline for control group	running for 30 km within within 6 weeks inducing OA<br><br>3. Non-running PNE group: only nicotine 2 mg/kg/d during GD 10-20<br><br>4. Non-running control group: saline 2 mg/kg/d during GD 10-20		Immunohistochemistry: expression of Col2a1, IGF-I, AKT1/2, IRS1 and SOX9 protein<br><br>ELISA: serum corticosterone and IGF-I concentrations	non-running group at PW 24; significantly increased in running PNE group compared with running control group at PW 24<br><br>3. Expression of Col2a1 protein: significantly decreased in both running and non-running PNE group at PW 24; significantly decreased in both control and PNE group after running compared with non-running group at PW 24<br><br>5. Expression of IGF-I, AKT1/2, and SOX9 protein: significantly reduced in both non-running and running PNE group at PW 24<br><br>6. Serum corticosterone concentrations: significantly increased in PNE group (603 ± 72 ng/ml) at GD 20<br><br>7. Expression of Col2a1 and SOX9 protein: significantly reduced in PNE group at GD 20<br><br>8. Expression of Col2a1 mRNA: significantly reduced in PNE group at GD 20<br><br>9. Expression of IGF-I, AKT1/2 protein: significantly reduced with nicotine at GD 20	susceptibility to OA in adult offspring.<br><br>2. Adult rat offspring with PNE are more susceptible to OA by the low-functional programming of IGF-I in fetal articular cartilage caused by the direct effect of nicotine via α4β2-nAChR and possibly by the indirect effects of maternal GC.	

Abbreviations: ABCA1, ATP-binding cassette transporter A1; ACAN, aggrecan; AKT1/2, serine–threonine protein kinase 1/2; ChIP assay, chromatin immunoprecipitation assay; Col2a1, α1 chain of type-II collagen; ECM, extracellular matrix; ELISA, enzyme-linked immunosorbent assay; GC, glucocorticoid; GD, gestational day; HC, hyaline cartilage; HDL-C, high-density lipoprotein cholesterol; H&E staining, hematoxylin and eosin staining; H3K9, lysine 9 of histone H3; IGF-I, insulin-like growth factor I; IRS1, insulin receptor substrate1; LDL-C, low-density lipoprotein cholesterol; LXR β: liver X receptor β; OARSI scores, Osteoarthritis Research Society International scores; PNE, prenatal nicotine exposure; PPARγ, peroxisome proliferator-activated receptor γ; PW, postnatal week; Smad2/3, SMAD Family Member 2/3; SOX9, SRY-type high mobility group box 9; TGF-β, transforming growth factor-β; TGF-βR1, transforming growth factor-β receptor 1.

References

1. Deng Y, Li TQ, Yan YE, Magdalou J, Wang H, Chen LB. Effect of nicotine on chondrogenic differentiation of rat bone marrow mesenchymal stem cells in alginate bead culture. Biomed Mater Eng. 2012;22(1-3):81-87.
2. Henderson JS, Johnson RB. The effects of smokeless tobacco extract on bone nodule formation and mineralization by chick osteoblasts in vitro. Arch Oral Biol. 1995;40(7):615-621.
3. Lenz LG, Ramp WK, Galvin RJ, Pierce WM, Jr. Inhibition of cell metabolism by a smokeless tobacco extract: tissue and species specificity. Proc Soc Exp Biol Med. 1992;199(2):211-217.
4. Liu Y, Wu D, Song F, et al. Activation of alpha7 nicotinic acetylcholine receptors prevents monosodium iodoacetate-induced osteoarthritis in rats. Cell Physiol Biochem. 2015;35(2):627-638.
5. Ng TK, Huang L, Cao D, et al. Cigarette smoking hinders human periodontal ligament-derived stem cell proliferation, migration and differentiation potentials. Scientific reports. 2015;5:7828-7828.
6. Niemeyer P, Salzmann GM, Hirschmuller A, Sudkamp NP. [Factors that influence clinical outcome following autologous chondrocyte implantation for cartilage defects of the knee]. Z Orthop Unfall. 2012;150(1):83-88.
7. Shaito A, Saliba J, Husari A, et al. Electronic Cigarette Smoke Impairs Normal Mesenchymal Stem Cell Differentiation. Sci Rep. 2017;7(1):14281.
8. Teng P, Liu Y, Dai Y, Zhang H, Liu WT, Hu J. Nicotine Attenuates Osteoarthritis Pain and Matrix Metalloproteinase-9 Expression via the alpha7 Nicotinic Acetylcholine Receptor. J Immunol. 2019;203(2):485-492.
9. Tie K, Tan Y, Deng Y, et al. Prenatal nicotine exposure induces poor articular cartilage quality in female adult offspring fed a high-fat diet and the intrauterine programming mechanisms. Reprod Toxicol. 2016;60:11-20.
10. Tie K, Wu M, Deng Y, et al. Histone hypo-acetylation of Sox9 mediates nicotine-induced weak cartilage repair by suppressing BMSC chondrogenic differentiation. Stem Cell Res Ther. 2018;9(1):98.
11. Tie K, Zhang X, Tan Y, et al. Intrauterine low-functional programming of IGF1 by prenatal nicotine exposure mediates the susceptibility to osteoarthritis in female adult rat offspring. Faseb j. 2016;30(2):785-797.
12. Wahl EA, Schenck TL, Machens H-G, Egaña JT. Acute stimulation of mesenchymal stem cells with cigarette smoke extract affects their migration, differentiation, and paracrine potential. Scientific reports. 2016;6:22957-22957.
13. Xie Z, Zhao Z, Yang X, et al. Prenatal nicotine exposure intergenerationally programs imperfect articular cartilage via histone deacetylation through maternal lineage. Toxicol Appl Pharmacol. 2018;352:107-118.
14. Yang X, Qi Y, Avercenc-Leger L, et al. Effect of nicotine on the proliferation and chondrogenic differentiation of the human Wharton's jelly mesenchymal stem cells. Biomed Mater Eng. 2017;28(s1):S217-s228.
15. Ying X, Cheng S, Shen Y, et al. Nicotine promotes proliferation and collagen synthesis of chondrocytes isolated from normal human and osteoarthritis patients. Mol Cell Biochem. 2012;359(1-2):263-269.

16. Ying X, Zhang W, Cheng S, et al. Nicotine-induced chondrogenic differentiation of human bone marrow stromal cells in vitro. Knee Surg Sports Traumatol Arthrosc. 2012;20(11):2329-2336.
